# Effect of 28 days of creatine ingestion on muscle metabolism and performance of a simulated cycling road race

**DOI:** 10.1186/1550-2783-7-26

**Published:** 2010-07-07

**Authors:** Robert C Hickner, David J Dyck, Josh Sklar, Holly Hatley, Priscilla Byrd

**Affiliations:** 1 Department of Exercise and Sport Science, Human Performance Laboratory, East Carolina University, Greenville, USA; 2 Department of Physiology, East Carolina University, Greenville, USA; 3 Department of Human Biology and Nutritional Sciences, University of Guelph, Ontario, Canada

## Abstract

**Purpose:**

The effects of creatine supplementation on muscle metabolism and exercise performance during a simulated endurance road race was investigated.

**Methods:**

Twelve adult male (27.3 ± 1.0 yr, 178.6 ± 1.4 cm, 78.0 ± 2.5 kg, 8.9 ± 1.1 %fat) endurance-trained (53.3 ± 2.0 ml* kg^-1^* min^-1^, cycling ~160 km/wk) cyclists completed a simulated road race on a cycle ergometer (Lode), consisting of a two-hour cycling bout at 60% of peak aerobic capacity (VO_2peak_) with three 10-second sprints performed at 110% VO_2 peak _every 15 minutes. Cyclists completed the 2-hr cycling bout before and after dietary creatine monohydrate or placebo supplementation (3 g/day for 28 days). Muscle biopsies were taken at rest and five minutes before the end of the two-hour ride.

**Results:**

There was a 24.5 ± 10.0% increase in resting muscle total creatine and 38.4 ± 23.9% increase in muscle creatine phosphate in the creatine group (*P *< 0.05). Plasma glucose, blood lactate, and respiratory exchange ratio during the 2-hour ride, as well as VO_2 peak_, were not affected by creatine supplementation. Submaximal oxygen consumption near the end of the two-hour ride was decreased by approximately 10% by creatine supplementation (P < 0.05). Changes in plasma volume from pre- to post-supplementation were significantly greater in the creatine group (^+^14.0 ± 6.3%) than the placebo group (^-^10.4 ± 4.4%; *P *< 0.05) at 90 minutes of exercise. The time of the final sprint to exhaustion at the end of the 2-hour cycling bout was not affected by creatine supplementation (creatine pre, 64.4 ± 13.5s; creatine post, 88.8 ± 24.6s; placebo pre, 69.0 ± 24.8s; placebo post 92.8 ± 31.2s: creatine vs. placebo not significant). Power output for the final sprint was increased by ~33% in both groups (creatine vs. placebo not significant).

**Conclusions:**

It can be concluded that although creatine supplementation may increase resting muscle total creatine, muscle creatine phosphate, and plasma volume, and may lead to a reduction in oxygen consumption during submaximal exercise, creatine supplementation does not improve sprint performance at the end of endurance cycling exercise.

## Background

Muscle creatine phosphate content has been shown to decline during prolonged exercise at 70% VO_2_max [1,2]. It is also well-established that dietary creatine supplementation can increase muscle creatine phosphate content and creatine phosphate resynthesis rates; thereby improving high-intensity intermittent exercise performance [3-6]. However, it is not known if creatine supplementation prior to exercise can elevate muscle total creatine and creatine phosphate content sufficiently to maintain muscle creatine phosphate content above those in a non-supplemented condition throughout prolonged endurance exercise. Increased muscle creatine phosphate content at the end of endurance exercise may improve performance of a final sprint to exhaustion at the end of endurance exercise because creatine phosphate is a major source of ATP for muscle ATP hydrolysis during short duration (< 30s) maximal-intensity efforts [7]. There are conflicting data as to whether or not creatine ingestion results in improved performance of prolonged exercise [8-12]. There have to date been five studies of the effects of creatine ingestion on performance of exercise lasting longer than 20 minutes. Three of these studies demonstrated improved performance of either continuous prolonged exercise (1 hour time trial) or of intermittent sprints following prolonged exercise [8-10]. Two other studies reported no change, or a decrement in performance following: a) a 25 kilometer cycling time trial interspersed with 15-second sprints [11] or b) a one hour time trial on a cycle ergometer [12]. Some of the studies were not double blind, randomized, or performed with a placebo; furthermore, muscle biopsies were obtained to document increased muscle creatine phosphate stores in only one of these previous studies. Exercise in these previous studies was performed following 5-7 days ingestion of 20 grams per day of a creatine supplement.

There is sufficient evidence that creatine ingestion of 20 grams per day over five days increases muscle creatine phosphate content and increases performance of repeated short bouts of high-intensity intermittent exercise [3,13-15]. Chronic, rather than short-term (less than one week), creatine supplementation is more commonplace in athletes, yet little is known of the effects of chronic creatine supplementation on muscle creatine phosphate levels and performance. There is only one published study demonstrating that ingestion of substantially less creatine over a longer period of time results in significant increases in muscle creatine phosphate content [16].

The purposes of the present investigation were therefore to determine if ingestion of 3 g/day of creatine monohydrate for 28 days would: 1) increase muscle creatine phosphate and total creatine content at rest and at the end of prolonged endurance exercise; and 2) increase sprint performance at the end of a prolonged bout of endurance exercise. The present study is unique in that it is the first double-blind study to monitor the effect of prolonged creatine supplementation at the level of the whole body, vascular compartment, and skeletal muscle.

## Methods

### Subjects

Twelve adult male (18-40 yr) endurance-trained (~160 km/wk) cyclists (Table 1) were studied before and after 28 days of ingestion of either 3 g/day creatine monohydrate (n = 6) or placebo (n = 6). The cyclists had been cycling at least 150 km/wk for the past year, and were familiarized with the cycle ergometer during testing of peak aerobic capacity and a 30-minute familiarization session the week prior to performance of the first endurance exercise test. Subjects had not been ingesting creatine or other dietary supplements other than a multivitamin and carbohydrate beverages for at least three months prior to the study as determined by questionnaire. The subjects were matched for body weight, percent body fat, VO_2_peak, and training distance cycled per week. The supplementation regime was administered in a double-blind fashion. The subjects participated in these investigations after completing a medical history and giving informed consent to participate according to the East Carolina University Human Subjects Committee.

**Table 1:** Subject Characteristics

Variables	Creatine Pre (n = 6)	Placebo Pre (n = 6)	Creatine Post (n = 6)	Placebo Post (n = 6)
Age (yr)	25.5 ± 1.6	29.0 ± 0.9	----	----
Height (cm)	177.2 ± 1.9	180.1 ± 2.1	----	----
Weight (cm)	78.1 ± 3.2	78.0 ± 4.1	80.1 ± 3.3*	78.7 ± 4.2
Percent fat (%) Hydrostatic	12.4 ± 1.1	9.6 ± 1.4	12.1 ± 1.4	9.5 ± 1.6
VO_2_max (L/min)	4.1 ± 0.3	4.2 ± 0.1	4.1 ± 0.3	4.3 ± 0.2
Distance per week (km)	156.9 ± 36.4	163.6 ± 27.1	---	---

*Different from pre (P < 0.05)

### Protocol

**Figure 1:**
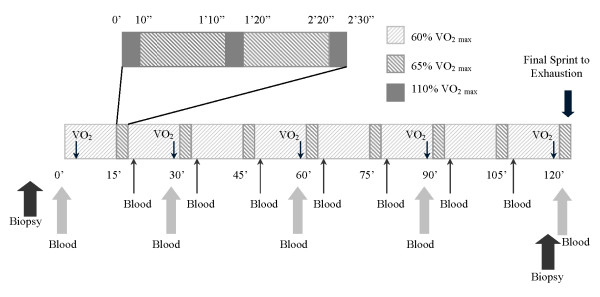
**Cyclists completed a 2-hour cycling bout on an electronically-braked cycle ergometer which consisted of 15 minutes of continuous exercise at 60% VO_2_peak followed by three, 10-second sprints performed at 110% VO_2_peak interspersed with 60 seconds cycling at 65% VO_2_peak**. This protocol was repeated eight times, for a total continuous exercise time of two hours. The final sprint was to exhaustion, with the duration of the final sprint used as the measure of performance. Muscle biopsies were obtained from the vastus lateralis of the quadriceps femoris muscle group immediately prior to, and five minutes prior to the end of, the cycling bout. A blood sample was obtained from an antecubital vein every 15 minutes. Oxygen consumption (VO_2_) was determined every 30 minutes.

Cyclists were tested for peak aerobic capacity and body composition at least 48 hours prior to performance of a two-hour bout of cycling on an electronically-braked cycle ergometer (LODE, Diversified Inc., Brea, CA). The cyclists also completed a diet record for the three days prior to, and the day of, their two-hour cycling session. The experimental protocol is presented in Figure 1. The 2-hour bout consisted of 15 minutes of continuous exercise at 60% VO_2_peak followed by three, 10-second sprints performed at 110% VO_2_peak interspersed with 60 seconds cycling at 65% VO_2_peak. This protocol was repeated eight times, for a total continuous exercise time of two hours. This protocol was designed to simulate a cycling road race that consists of multiple repeated sprints throughout the race to "drop" other cyclists from the lead group. The protocol was found to be the maximum intensity that this group of cyclists could maintain for the entire two hours as determined during pilot testing. The cyclists consumed water ad libitum throughout the ride. Immediately before and five minutes prior to the end of the ride a muscle biopsy was taken from the vastus lateralis of the quadriceps femoris muscle group. Blood samples (See Figure 1) were taken immediately prior to, during (immediately before and after each interval set), and immediately after the ride from an intravenous catheter placed in a forearm vein. The cyclists completed all testing described above twice, once before and once after 28 days of either three grams/day creatine or placebo ingestion. The second 2-hour cycling bout was performed at the same power outputs as was performed prior to supplementation. The only factor that changed was the time of the final sprint, which was performed to exhaustion. Total work performed during the final sprint was then calculated from the power output set on the cycle ergometer and the total time of the sprint. The cyclists maintained the same dietary and training regimen for the three days prior to the second two-hour cycling bout, and consumed the same amount of water during the second as the first two-hour cycling bout. The cyclists were also instructed not the change their training habits during the supplementation period.

### Body Composition and Anthropometric Determinations

Residual volume was determined by the oxygen dilution method as described by Wilmore [17]. Body density was determined by hydrostatic weighing, with percent body fat calculated using residual volume and body density using the equations of Brozek *et al*.[18]. Our coefficient of variation of test-retest for hydrostatic weighing is 8.1 ± 2.0%, which is approximately 1% body fat in individuals with approximately 10% fat.

### Peak Aerobic Capacity (VO_2_peak)

Peak aerobic capacity was determined on an electronically-braked cycle ergometer according to the American College of Sports Medicine guidelines. The test was incremental, beginning at 150 Watts and increasing exercise intensity by 50 Watts every three minutes. Respiratory gases were analyzed continuously and averaged over 20-second intervals using a Sensormedics 2900 Metabolic Measurement Cart (Anaheim, CA). The subjects exercised until they could no longer maintain a cadence of 40 revolutions per minute. Achievement of VO_2_peak was determined by attainment of two of the following criteria: 1) plateau in oxygen consumption with increased exercise intensity, 2) respiratory exchange ratio (RER) > 1.1, and 3) heart rate greater than age-predicted maximal heart rate. Our coeffient of variation of test-tetest is 4.1 ± 1.1% for cycling VO2max testing.

### Dietary creatine supplementation and nutritional assessment

Subjects kept a dietary log of everything ingested for the three days prior to, and the day of, their two-hour cycling session. The log was then analyzed using the nutritionist IV Diet Analysis computer software (version 4.1; First DataBank Corporation, San Bruno, CA). The cyclists were then instructed to consume a diet for the last three days of supplementation that was identical in composition, with the exception of the creatine or placebo supplement, to the diet ingested prior to supplementation. The subjects were instructed to ingest the supplement (three grams creatine monohydrate or placebo mixed in four ounces of water) once per day, in the evening with dinner, for 28 days. The last dose of the supplement was ingested 14 hours before the endurance cycling test. The placebo was a mixture of two grams condensed dry milk and one gram orange-flavored carbohydrate (Tang, Kraft foods). The creatine supplement was composed of three grams creatine monohydrate (EAS, Golden, CO) mixed with the contents used in the placebo drink.

### Blood sampling and analyses

Blood was drawn from an antecubital vein of subjects in a seated position 4 hours after their most recent meal. Every thirty minutes during the 2-hour cycling bout a 10 ml blood sample (five ml in an untreated test tube and 5 ml in an EDTA-treated tube) was drawn. Whole blood was used for determination of hematocrit and hemoglobin in triplicate. Plasma volume was then calculated from hemoglobin and hematocrit values at each time point [19]. Blood samples collected in EDTA-treated tubes were centrifuged at 2000 × g for ten minutes. The supernatant was drawn off and placed into microcentrifuge tubes for subsequent analyses. Plasma samples were analyzed for lactate and glucose in duplicate using a YSI 2300 STAT Plus Glucose Analyzer (Yellow Springs, OH). Plasma lactate data were converted to whole blood lactate data using a correction factor (1.05) to account for lactate in red blood cells.

### Muscle biopsy

Muscle biopsies (~100 mg) were obtained percutaneously under local anesthesia (2-3 cc 1% lidocaine) from the vastus lateralis of the quadriceps femoris muscle group at rest immediately prior to the cycling bout and five minutes prior to the end of the two-hour cycling bout. It was necessary for the cyclist to stop cycling for approximately 20 seconds for the second biopsy procedure and bandaging. The muscle biopsy samples were immediately (< 2 seconds from the time of excision) frozen in liquid nitrogen. A 5-10 mg piece of muscle was cut while frozen from the original piece of muscle and was mounted in tragacanth-OCT (Miles, Elkhart, IN) mixture and stored at -80°C for subsequent fiber type analysis by histochemistry [20]. This method may have resulted in more freeze-fracturing than had the muscle been mounted for histochemistry been frozen slowly in isopentane; however, the quick freeze of the sample was imperative for analyses of high-energy phosphates. The remaining sample was stored under liquid nitrogen until subsequently lyophilized overnight. Samples were then dissected free of blood and connective tissue and partitioned for subsequent analysis of adenosine triphosphate (ATP), creatine phosphate (CP), creatine (Cr), and glycogen concentration using spectrophotometric methods as previously described [21].

### Side effects

Subjects filled out a health questionnaire before and after supplementation to determine if any adverse side effects were encountered. Included in the list of possible side effects were questions of muscle cramping, chest pain, fatigue, upper-respiratory and auditory problems, autoimmune reactions, gastrointestinal difficulties, syncope, joint discomfort, appetite, headache, memory, stress and mood changes.

### Statistics

For each variable a two-way [treatment (creatine or placebo) * time (pre and post supplementation)] repeated measures ANOVA. ANCOVA was performed using pre data as a covariate for hemoglobin, hematocrit, muscle total creatine, and muscle lactate analyses because of differences between creatine and placebo groups prior to supplementation. When significant results were found, Newman-Keuls' post hoc analysis was used.

## Results

Subject characteristics (age, height, body mass, percent fat, VO_2_peak, and training mileage) are presented in Table 1. Body mass was 2.0 kg higher after supplementation than before supplementation (P < 0.05). There were no differences between creatine and placebo groups for all other descriptive variables.

### Sprint time

**Figure 2:**
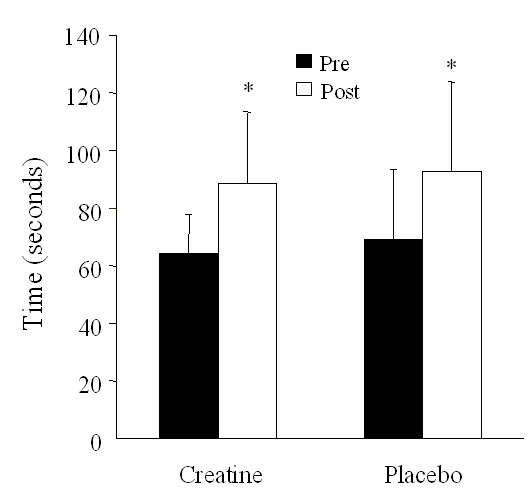
**Mean duration of the final sprint following approximately 2-hours of cycling performed before and at the end of 28 days of dietary supplementation (3 g/day creatine; n = 6 or placebo; n = 6) in young trained cyclists**. Data are presented as mean ± SEM.

The final sprint times prior to supplementation were 64.4 ± 13.5 and 69.0 ± 24.8 seconds in the creatine and placebo groups, respectively (Figure 2). There was a main effect (*P *< 0.05) for sprint time pre to post supplementation, in that creatine and placebo groups both increased final sprint times following supplementation by approximately 25 seconds.

### Power output

The power output for the final sprint prior to supplementation was 23,459 ± 6,430 and 19,509 ± 2,969 joules in the creatine and placebo groups, respectively. There was a main effect (*P *< 0.05) for power output pre to post supplementation, in that creatine and placebo groups both increased final power output after supplementation by approximately 33%. The power output for the final sprint after supplementation was 30,811 ± 10,198 and 26,599 ± 3,772 joules in the creatine and placebo groups, respectively.

### Respiratory exchange ratio (RER) and oxygen consumption (VO_2_)

**Figure 3:**
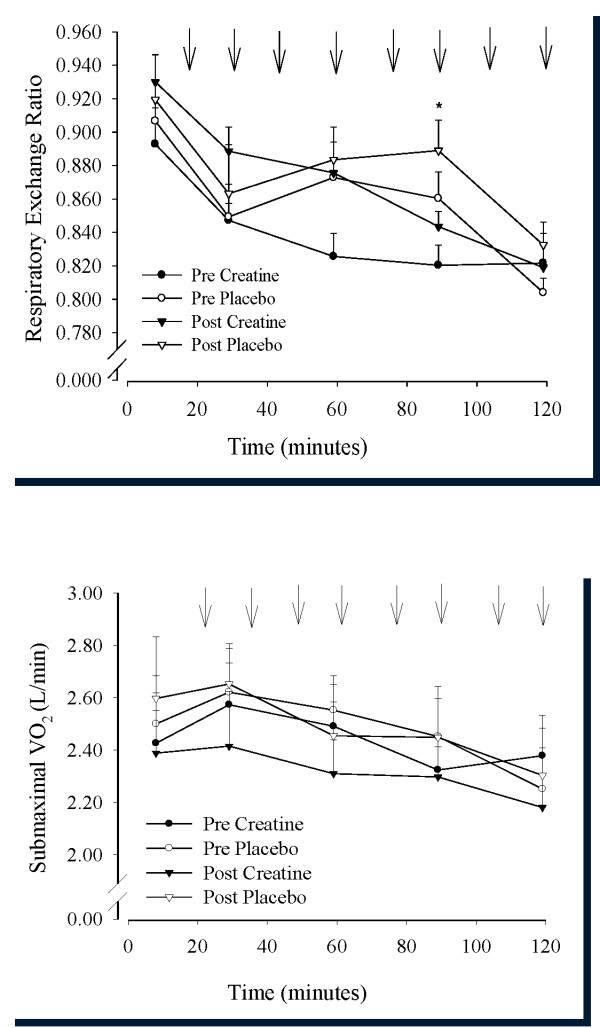
**a and b - Mean respiratory exchange ratio (RER; Figure 3a) and submaximal oxygen consumption (Figure 3b) during approximately 2-hours of cycling performed before and at the end of 28 days of dietary supplementation (3 g/day creatine; n = 6 or placebo; n = 6) in young trained cyclists**. Arrows denote sprint bouts. Data are presented as mean ± SEM. * different from creatine (*P *< 0.05). ** Submaximal oxygen consumption lower post than pre supplementation at 117 minutes.

Mean RER values during the two-hour cycling bout were similar in both groups prior to supplementation and decreased from approximately 0.91 to 0.82 from 7 to 119 minutes of the cycling bout. RER during the ride was not affected by the type of supplementation, in that both creatine and placebo groups demonstrated a decline in RER over time (Figure 3a). There was an interaction in submaximal VO_2 _(Figure 3b) at minute 119 of the cycling bout due to the lower oxygen consumption after than before creatine ingestion and the higher oxygen consumption after than before placebo ingestion.

### Blood glucose and lactate

**Figure 4:**
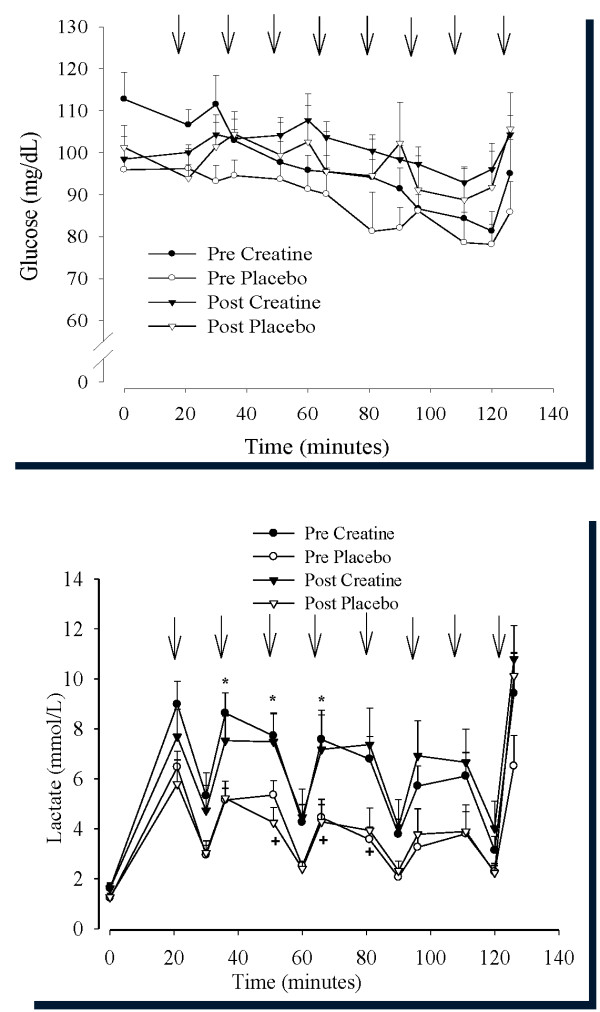
**a and b - Mean plasma glucose (Figure 4a) and blood lactate (Figure 4b) during approximately 2-hours of cycling performed before and at the end of 28 days of dietary supplementation (3 g/day creatine; n = 6 or placebo; n = 6) in young trained cyclists**. Arrows denote sprint bouts. Data are presented as mean ± SEM. * pre creatine different from pre placebo. ^+^Post placebo different from post creatine. All values were elevated from 0 minutes (*P *< 0.05).

There was a main effect for plasma glucose pre- to post-supplementation (P < 0.05; Figure 4a) resulting from higher plasma glucose concentrations after than before supplementation in both creatine and placebo groups. Blood lactate was higher in the creatine group than the placebo group during the 2-hour cycling bout both before and after supplementation (Figure 4b). There was a four- to six-fold increase in blood lactate from rest to the end of each set of sprints, although blood lactate was only two- to three-fold higher than resting at the end of each 15-minutes of cycling at 60% VO_2_peak. Blood lactate was not different after, compared to before, supplementation in either creatine or placebo groups.

### Hemoglobin, hematocrit, and plasma volume

**Figure 5:**
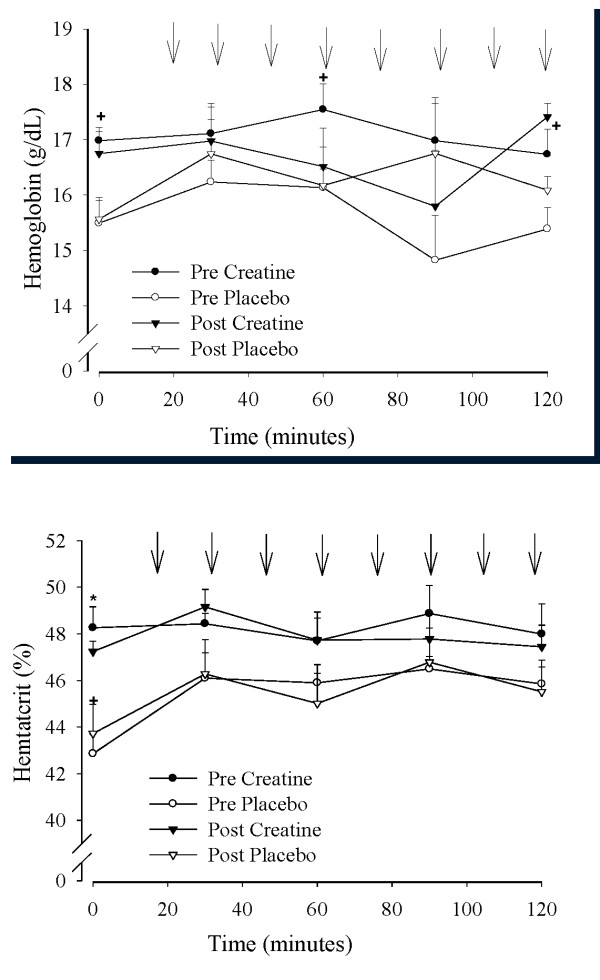
**a and b - Mean hemoglobin (Figure 5a) and hematocrit (Figure 5b) during approximately 2-hours of cycling performed before and at the end of 28 days of dietary supplementation (3 g/day creatine; n = 6 or placebo; n = 6) in young trained cyclists**. Arrows denote sprint bouts. Data are presented as mean ± SEM. ^+^pre creatine different from pre placebo.

Hemoglobin and hematocrit were approximately 10% higher in the creatine group (48% and 17 mg/dl) than placebo group (43.5% and 15.5 mg/dl) both before and after supplementation: there was no effect of supplementation on either variable (Figures 5a and 5b). The changes in hemoglobin and hematocrit were reflective of changes in resting plasma volume from pre- to post-supplementation of +4.7 ± 4.7% and +0.5 ± 2.1% in the creatine and placebo groups, respectively (*P *= N.S.). Changes in plasma volume from pre- to post-supplementation were significantly greater in the creatine group (+14.0 ± 6.3%) than the placebo group (-10.4 ± 4.4%; *P *< 0.05) at 90 minutes of exercise.

### Muscle creatine, total creatine, creatine phosphate, and adenosine triphosphate

**Figure 6:**
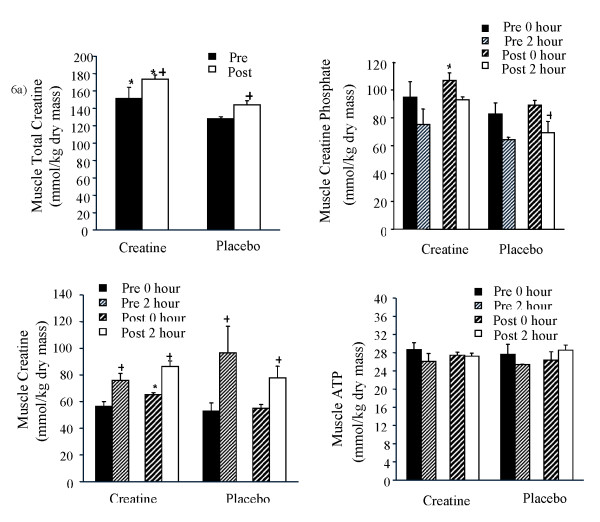
**a-d. Mean muscle total creatine (Figure 6a), creatine phosphate (Figure 6b), creatine (Figure 6c), and muscle ATP (Figure 6d) during approximately 2-hours of cycling performed before and at the end of 28 days of dietary supplementation (3 g/day creatine; n = 6 or placebo; n = 6) in young trained cyclists**. Data are presented as mean ± SEM. *creatine different from corresponding placebo. + post different from pre.

Resting muscle total creatine concentrations (Figure 6a) were higher in the creatine than placebo groups both before and after supplementation, although muscle total creatine increased following supplementation in both groups. When calculating the increase in muscle creatine for each individual pre- to post-supplementation, the mean increase in muscle total creatine was 24 ± 11% in the creatine group and 15 ± 3% in the placebo group (p = N.S.).

Muscle creatine phosphate (CP; Figure 6b) at rest was not different between creatine and placebo groups prior to supplementation, although muscle CP was higher following supplementation in the creatine than placebo group (P < 0.05). When calculating the increase in muscle CP during supplementation on an individual basis, the increase in resting muscle CP was 38 ± 27% in the creatine group and 14 ± 11% in the placebo group. There was a significant drop in muscle CP by the end of the two-hour ride after supplementation in the placebo group (P < 0.05), although this drop was not as evident in the creatine group (Figure 6b). There was no correlation between the change in muscle creatine phosphate and the change in sprint performance from pre- to post-supplementation.

Resting muscle creatine concentration (Figure 6c) was increased by supplementation in the creatine group (P < 0.05). Muscle creatine concentration was increased (P < 0.05) to a similar extent during the two-hour cycling bout in creatine and placebo groups.

With respect to muscle ATP content (Figure 6d), there was a significant main effect for time, in that there was a drop in muscle ATP over the two-hour cycling bout prior to supplementation that was not seen following supplementation in either creatine or placebo groups. There was therefore no effect of supplementation on muscle ATP content in resting or exercising muscle.

### Muscle lactate and glycogen

**Figure 7:**
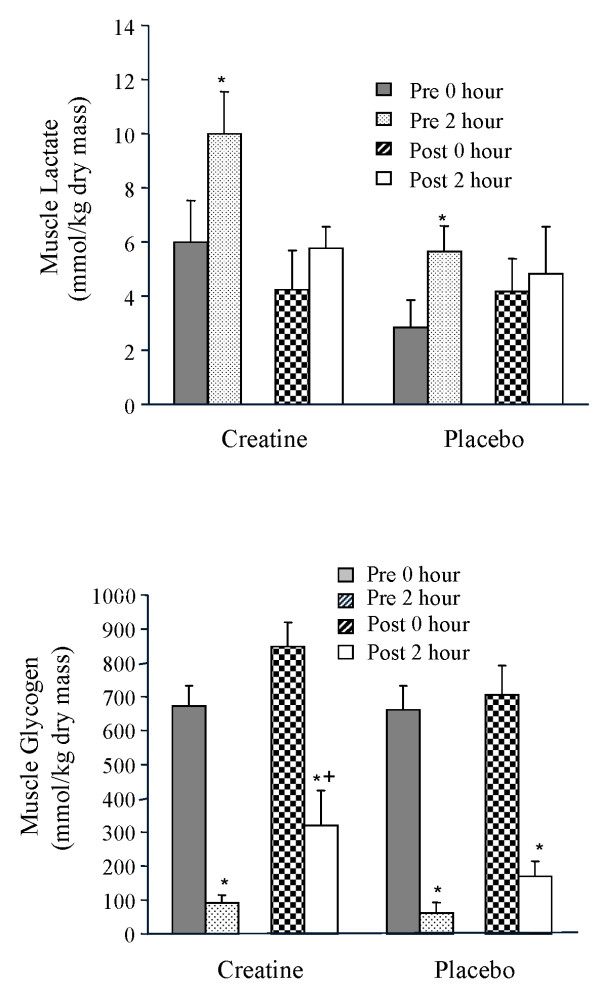
**a and b. Mean muscle lactate (Figure 7a) and muscle glycogen (Figure 7b) during approximately 2-hours of cycling performed before and at the end of 28 days of dietary supplementation (3 g/day creatine; n = 6 or placebo; n = 6) in young trained cyclists**. Data are presented as mean ± SEM.

Muscle lactate (Figure 7a) concentration increased for both creatine and placebo groups from rest to the end of the two-hour cycling bout before supplementation; however, after supplementation both groups exhibited less of an increase in muscle lactate during the two-hour cycling bout. Muscle glycogen content (Figure 7b) was reduced (*P *< 0.05) by approximately 600 mmol/kg dry mass both before and after supplementation in creatine and placebo groups. After supplementation, muscle glycogen content at the end of the two-hour ride was higher in the creatine than placebo group (*P *< 0.05) due to the higher resting muscle glycogen content after supplementation in the creatine than placebo group.

### Muscle fiber composition

Fiber type percentage in the creatine group was 46.8 ± 3.6, 42.7 ± 2.4, and 10.5 ± 2.5% for type I, type IIa, and type IIb fibers, respectively. Fiber type percentage in the placebo group was not different from that of the creatine group, with fiber type percentages of 42.5 ± 2.3, 48.7 ± 3.8, and 8.5 ± 3.0% for type I, type IIa, and type IIb fibers, respectively. Type I fiber percentage was correlated with muscle total creatine (r = 0.62, P < 0.05) and muscle creatine phosphate (r = 0.65, P < 0.05). Fiber type percentage was not significantly correlated with sprint performance time, nor with the change in muscle creatine concentration from pre- to post-supplementation.

#### Side effects

Regarding side effects (data not shown), two of the 12 subjects reported experiencing muscle cramps at rest following supplementation. There were no reports of muscle cramping prior to supplementation. Both of the subjects who reported muscle cramping following supplementation were in the creatine group. There were no other reports of side effects (chest pain, fatigue, upper-respiratory and auditory problems, autoimmune reactions, gastrointestinal difficulties, syncope, joint discomfort, appetite, headache, memory, stress and mood changes) that were unique to the creatine supplementation.

## Discussion

The present study is unique in that it is the first double-blind study to monitor the effect of prolonged creatine supplementation at the level of the whole body, vascular compartment, and skeletal muscle. The performance data presented indicate that total time of a sprint to exhaustion at a constant power output following two hours of variable-intensity cycling is not influenced by 28 days of low-dose dietary creatine monohydrate supplementation. Sprint time, and therefore total power output, in the creatine group was not improved to a greater extent than that seen in the placebo group. Engelhardt et al. [8] and Vandeburie et al. [10] studied cyclists and triathletes consuming 6 g and 25 g creatine, respectively, per day for five days. These previous studies demonstrating an increased power output during alternating intensity, endurance exercise following creatine supplementation were different from the present study in a number of ways. In the study by Engelhardt et al.[8], 12 triathletes cycled for 30 minutes at 3 mmol/l blood lactate followed by ten 15-second intervals at 7.5 Watts/kg interspersed with 45 seconds rest, a two-minute rest, ten more 15-second intervals, and another 30-minute cycling bout at 3 mmol/l blood lactate. The triathletes were able to generate 18% more power after than before creatine supplementation during the intervals. The subjects in the study, however, were not blinded as to treatment, with each subject undergoing the creatine cycling bout after the non-supplemented bout. Our study participants were blind to treatment or placebo, and performed a continuous sprint to exhaustion at a constant power output, rather than variable power during intervals in the study by Engelhardt et al.[8]. In another cycling study demonstrating positive effects of creatine supplementation during timed intervals at maximal intensity, Vandeburie et al. studied twelve elite cyclists in a double-blind fashion [10]. Vandeburie et al. allowed up to three minutes rest between a standardized 2.5 hr cycling bout and five, 10-second maximal intensity sprints that were used to gauge performance. Active recovery performed at 0.5 kg resistance was allowed for two minutes between each sprint. Although the cyclists were able to perform at 8-10% greater power outputs during the five 10-second sprints following creatine ingestion than following placebo ingestion, the three-minute recovery following the endurance ride may have influenced the results. It should also be noted that there was no difference in cycling time (approximately 10 minutes) for a cycling bout to fatigue performed at 4 mmol/l lactate threshold immediately at the end of the standardized endurance ride. A study by Rico-Sanz and Marco [9] also demonstrated improved performance (+6.5 minutes) in seven cyclists following creatine ingestion (20 g/day for 5 days) compared to seven cyclists consuming placebo. Performance in this study was measured as time to exhaustion (approximately 30 minutes) during alternating intensity exercise at 30% and 90% of maximal power output. The intensity and intermittent nature of the alternate-intensity cycling performance measure to exhaustion, as well as the high-dose supplementation regime in the study by Rico-Sanz and Marco was clearly different from our low-dose supplementation study with a performance measure of timed sprint to exhaustion at a constant power output. Muscle biopsy data, used to verify increases in muscle creatine phosphate content, are lacking in all of the studies described above, although blood analysis demonstrated a significantly higher plasma creatine and creatinine following supplementation in the study by Engelhardt et al. [8]. The primary difference between the present study, demonstrating no improved performance, and past studies, demonstrating improved cycling performance, is likely the type of performance measure: sprint to exhaustion at a constant power output in the present study as compared to interval-type performance at self-paced intensity in other studies.

The lack of effect of creatine supplementation on performance in the present study is similar to the findings of Godly et al. [11] and Myburgh et al.[12], published only in abstract form. Godly et al. detected no greater improvement in performance in eight cyclists consuming creatine (7 grams/day for 5 days) compared to eight cyclists who consumed placebo. Both groups were tested before and after the 5-day blinded supplementation period. The well-trained cyclists sprinted 15 seconds every four kilometers of a 25 km time trial performed in the laboratory on their own bikes [11]. Myburgh et al. [12] also detected no difference in one-hour time trial after seven days of supplementation at 20 g/day. Thirteen cyclists were tested before and after the supplementation period, with seven cyclists ingesting creatine and six ingesting placebo. These data conflict with past reports of positive benefits of creatine ingestion on endurance performance, and indicate that there is no consensus as to the effect of creatine supplementation on endurance performance of continuous or variable-intensity cycling.

The potential benefits of creatine supplementation include enhanced muscle creatine phosphate and muscle glycogen content, increased plasma volume, and alterations in substrate selection and oxygen consumption. Although there were positive effects of this low-dose creatine compared to placebo supplementation with respect to resting muscle creatine phosphate and glycogen content, as well as increased plasma volume and reduced submaximal oxygen consumption during exercise, there was no greater improvement in sprint performance in the creatine than placebo group.

There have been only two studies of creatine supplementation other than the present study reporting oxygen consumption during endurance exercise. Rico-Sanz and Marco [9] demonstrated an increased oxygen consumption following creatine ingestion when cyclists cycled at 90% of maximal power output. In contrast, we detected an interaction of treatment (creatine and placebo) and time (pre and post supplementation) for submaximal oxygen consumption near the end of the cycling bout in the present study, indicating that creatine supplementation results in lower submaximal oxygen consumption when cycling at 60% VO_2_peak. Differences in intensity and duration of the protocol may account for the discrepant findings of the current study and that of Rico-Sanz and Marco. Englehardt et al. [8] also reported submaximal oxygen consumption data, and found no effect of creatine supplementation on oxygen consumption during cycling at 3 mmol/l blood lactate. In the present study, submaximal oxygen consumption was 8-9% lower following creatine supplementation than following placebo near the end of two hours of cycling (*P *< 0.05), although the cause of this reduced oxygen consumption is unknown.

No previous studies of creatine supplementation and endurance exercise have contained reports of respiratory exchange ratio. We found no effect of supplementation on respiratory exchange ratio, suggesting that creatine supplementation does not alter fuel selection. There was also no difference between creatine and placebo groups in the change in muscle glycogen during the cycling bout. There was a higher muscle glycogen concentration five minutes prior to the end of exercise in the post-creatine cycling bout compared to the post-placebo cycling bout, but this was likely due to the slightly elevated muscle glycogen content prior to the post-supplementation exercise in the creatine group.

The vast majority of previous studies of creatine supplementation have used a five to ten day supplementation at 20 g/day. Hultman et al. [16] demonstrated that the high loading phase of creatine is not necessary if a longer supplementation period (28 days) is used. Their protocol of three g/day for one month had not been replicated prior to the current study. We have found that 28 days of creatine supplementation at three g/day increases muscle creatine phosphate to levels above a placebo group post supplementation. The increases in muscle creatine phosphate and total creatine were of similar magnitude (approx. 10 and 20 mmol/kg, respectively) to those demonstrated by Hultman et al. [16]. However, there also appeared to be increases, though not significant, in our placebo group of 5 mmol/kg and 10 mmol/kg and for creatine phosphate and total creatine, respectively. These data, in combination with our performance data demonstrating an increased performance that was not dependent upon the type of supplementation (creatine or placebo), highlight the importance of using a placebo group and a double-blind protocol. Although Hultman et al. included a placebo group in their study design, they did not take muscle biopsies from the control group.

## Conclusions

The present data support the findings of Hultman et al. [16] with respect to increases in muscle creatine phosphate with creatine supplementation at 3 g/day for 28 days. The creatine supplementation was also associated with higher pre-exercise body weight as well as higher muscle glycogen concentration and plasma volume near the end of two hours of cycling after creatine supplementation compared to placebo. It can be concluded that 28 days of creatine supplementation increased resting muscle creatine phosphate, muscle glycogen content and plasma volume during exercise. The creatine supplementation was not different from placebo in improving performance of a sprint to exhaustion at the end of a two-hour cycling bout interspersed with eight sets of three 10-second sprints.

## Abbreviations

ANOVA: Analysis of variance; ANCOVA: Analysis of covariance; ATP: Adenosine triphosphate; CP: Creatine phosphate; CR: Creatine; RER: Respiratory exchange ratio; VO_2_peak: Peak aerobic capacity.

## Declaration of Competing interests

The authors declare that they have no competing interests.

## Authors' contributions

RCH participated in protocol design, conduct of the study, data analysis and manuscript preparation. DD participated in protocol design, sample analyses and manuscript preparation. JS participated in data collection, sample analysis and manuscript review. HH participated in data collection, sample analysis and manuscript review. PB participated in participant recruitment data collection, and manuscript review. All authors read and approved the final version of the manuscript
